# Short Sight and Its Causes

**Published:** 1889-02-09

**Authors:** 


### Short Sight and Its Causes.

The world is very old, and our nation is very old, and so we, its inhabitants, are very old, even the children among us; and consequently our sight is failing. This seems to be the inference to be drawn from the recent correspondence in the *Standard* on the subject of defective vision in children. One of the surgeons to the Western Ophthalmic Hospital, was the first to raise the alarm, he having been struck, to use his own words, "by the enormous number of children who are brought to the out-patient department in quest of assistance with regard to vision"; and his suggestion for the remedy of this national disaster was that the Government should appoint ophthalmic surgeons to examine and report on the vision of children before they enter Board Schools.

After this came a leader in the *Standard*, and then more and yet more letters. Myopia is not such an interesting subject as Marriage to the letter-to-the-editor writer; but there was a very fair flow of ink and opinions, both from those who knew something about the subject, and those who ought to have known that they didn't. Of course, there were the usual explanations of the supposed increase of short-sightedness. Gas was blamed, and so was paraffin, and the feeble rushlight and modest "dip" of bygone ages were praised at the expense of their modern supplanters. Then suspicion fell on the supposed bad type and cheap paper of modern books. Is this fair? In the first place, the books of past generations were infinitely worse printed, and on worse paper than any that our children are forced to read; and, as a matter of fact, the large majority of school-books are infinitely superior in these respects to the average newspaper. Indeed, if bad type and cheap paper were important factors in the development of short-sight, the readers in newspaper offices ought all to suffer in a marked degree. There must be some other cause than this, and we will not lay the responsibility of encouraging myopia on the shoulders of that over-burdened scapegoat, the School Board.

But it would be well to inquire if the increase of myopia is as serious as some of the correspondents of the *Standard* seem to think. That spectacled children, young men, and maidens, are more frequently seen than of yore none will deny, but the cause of this very probably is that more attention is paid to ophthalmic diseases now than formerly. We now take to the doctor slight defects of vision which our fathers endured meekly till they ended in total blindness. Oculists are common, and, besides special hospitals for the treatment of weakness and disease of the eye, all our large hospitals have ophthalmic departments. This explanation is not our own; it is given by one of the writers to the *Standard*, who signs himself "Oculus," and who is evidently a professional surgeon. "Oculus" also reminds the excited ones who applied the principle of heredity in its narrowest bearings to the transmission of myopia, that there is another evolutionary principle which is equally sure, though not so popular, that, namely, of reversion to the normal type. There is really no imminent danger of our becoming a nation of moles.

We will not deny that there is a certain amount of myopia prevalent, especially in towns and among the studious classes of the community. A man of science, having fatigued his eyes by peering through a microscope, finds his vision impaired, and straightway continues the injurious strain by putting on a pair of spectacles; wherein he proves himself an unscientific man, ignorant or oblivious of the fact that rest should come after exertion, to each organ as well as to the whole organism. Or a young lady strains her eyes over crewel work, or in studying the subjects set for the Cambridge examination, according to her tastes; and because she wants to finish the embroidery, or pass the examination in the shortest possible time, she buys a pair of spectacles. Or—we suggest this diffidently—she thinks that a pince nez is becoming, that it adds piquancy to her face, and so accredits herself with a slight degree of myopia, invests half-a-crown in the purchase of an eye-glass, and sixpence more for a cord to hang it by, and then goes about the streets, unconscious that she gives the impression that the national sight is failing. It is possible indeed that the wearing of glasses may weaken her eye-sight, as "coddling" will weaken any organ, and in the end she will no more be able to do without them than without the morning cup of tea her grandmother would have scorned to indulge in. Even spectacles may become a luxury.

It may be said, however, that they are not a luxury likely to be desired by children. That is uncertain. The instructive anecdote which tells how Tom Sawyer got all his playfellows to help him in painting his aunt's gate has a certain bearing on this point. Children will do anything if they think it is a privilege, and it is not at all incredible that Dick will do Jim's exercise for him, if as a reward he is allowed to make his eyes water by wearing Jim's spectacles for half an hour. By this means Dick has a good chance, though he does not know it, of qualifying for spectacles of his own.

In fact, children should not, if it can be avoided, be given spectacles to wear. These do not always sit straight on the rétroussé nose of youth. The patient looks over them or under them, or through one glass only, all of which things tend to aggravate rather than relieve disease of the eye. Where it is at allpossible attention should be given first to resting and then to strengthening the sight. A certificate of visual defect should be enough to give any child the right to escape from any task involving special strain on the eyes, and in such cases school-books printed in extra large type might be provided. Simultaneously with these preventive measures, attention should be given to strengthening the sight, by training the child to look at and observe things at a distance. The proverbially long sight of sailors is due to their constant exercise of the utmost powers of vision. "M. R. C. S." also writes in the *Standard*: "It was the practice in Russia some years ago to endeavour to cure, or at all events to alleviate this defect (short-sightedness) by placing the reading-lesson as far from the eyes as it was capable of being read, the patient not being permitted to approach any nearer. It was soon found that the eye became accustomed to it, and by degrees the distance was increased, and the nearsightedness diminished."

This is, of course, not a cure that can be recommended for every kind of defect of vision, but for a moderate degree of myopia it is a wholesome tonic, better by far than that acceptance of the weakness as inevitable which is too common at present. The best cure is that which strengthens rather than that which supplements the weak sight. The statistics of ophthalmology are as yet too recent and imperfect to justify us in saying with any assurance whether or not short-sightedness is increasing; but in any case a little common sense applied to the development of preventive and corrective measures will do much to modify the evil, and free the already burdened ratepayer from having to keep up a board of ophthalmic examiners, or any other elaborate and dubiously useful machinery.

